# Importance of appropriate pharmaceutical management in pregnant women with ulcerative colitis

**DOI:** 10.1186/1756-0500-6-210

**Published:** 2013-05-25

**Authors:** Masaki Ujihara, Takafumi Ando, Kazuhiro Ishiguro, Osamu Maeda, Osamu Watanabe, Yutaka Hirayama, Kazuhiro Morise, Keiko Maeda, Masanobu Matsushita, Ryoji Miyahara, Naoki Ohmiya, Yuji Nishio, Takeo Yamaguchi, Jun-ichi Haruta, Kenji Ina, Hidemi Goto

**Affiliations:** 1 Department of Gastroenterology and Hepatology, Nagoya University Graduate School of Medicine, Nagoya 466-8550, Japan; 2 Department of Gastroenterology, Meitetsu Hospital, Nagoya 451-8511, Japan; 3 Department of Gastroenterology, Nagoya First Red Cross Hospital, Nagoya 453-8511, Japan; 4 Department of Medical Oncology, Nagoya Memorial Hospital, Nagoya 468-0011, Japan

**Keywords:** Ulcerative colitis, Pregnancy, Clinical course, Treatment

## Abstract

**Background:**

Ulcerative colitis (UC) often occurs in women of childbearing age. Compared to Western countries, however, few studies have investigated the impact of UC on the progress of pregnancy in Asian populations.

**Methods:**

We retrospectively examined 91 pregnancies in 64 patients with UC experienced at our hospital and related institutions from 1991 to 2011, focusing on the relationship between the progression of UC during pregnancy, progress of the pregnancy itself, and the treatment of UC.

**Results:**

In 80 of 91 pregnancies the patient had already been diagnosed with UC at the time she became pregnant, of whom 31 (38.8%) experienced exacerbation during pregnancy. Regarding severity, moderate or severe active-stage disease during pregnancy was seen in 13.7% of those who had been in remission at the onset of pregnancy versus 58.6% of those who had been in the active stage at onset (OR 8.9: 95%CI 3.0~26.4; P<0.01). The incidence of miscarriage or abortion was 9.8% in pregnancies in which UC was in remission at onset versus 31% in those in which it was in the active stage at onset (OR 4.1: 95%CI 1.2~13.9; P=0.02). Among patients, 62.5% were receiving pharmaceutical treatment at onset of pregnancy. Exacerbation during pregnancy occurred in 26.5% of the group who continued to receive the same treatment during pregnancy versus 56.3% of those with a dose decrease or discontinuation after onset (OR 3.6: 95%CI 1.0~12.4; P=0.04).

**Conclusions:**

UC patients wishing to conceive should do so when in remission and continue appropriate pharmaceutical treatment during pregnancy.

## Background

Recent reports suggest that the number of patients with ulcerative colitis (UC) has continued to increase steadily in East Asia [1-6]. Onset of UC typically occurs during adulthood, affecting individuals during their reproductive years. Patients with UC want to know whether disease activity and medical treatment exert any influence on their fertility, pregnancy, and babies. While several reports on the effects of UC on the course of pregnancy have appeared from Europe and the US [7-12], only a few studies have been reported from Asia [13,14]. In particular, information regarding disease activity and medication is scarce, because these investigations are not easily conducted. Vermeire et al. [15] suggested that clinicians should ensure therapy compliance by discussing with patients the need for drug therapy to maintain remission, and should mention that most drugs for inflammatory bowel disease (IBD) are compatible with pregnancy. ECCO [10] also reported that in the majority of patients, the maintenance of remission with medical treatment outweighs the potential risks of adverse drug effects.

At present, the relationship between pregnancy and UC has not been well investigated in Asian countries. Although Western guidelines based on data obtained from UC patients in Western countries may be useful in the management of UC patients in Japan, clinical conditions of UC in Asians show important differences from those in Caucasians [16-18]. For example, the severity of UC in Asian patients is relatively mild, with few cases requiring surgery and low mortality rates [16]. Furthermore, genetic backgrounds and environmental factors influencing UC differ between Asian and western countries. These differences highlight the need for research into the interrelation of UC course, treatment, and pregnancy in Asian populations.

To better characterize the relationship of pregnancy and UC in Japan, we have focused on disease activity and medical treatment during pregnancy. Here, we conducted a retrospective clinical examination of the course of pregnancies experienced by patients with UC at our hospital and related facilities.

## Methods

### Subjects

**Figure 1:**
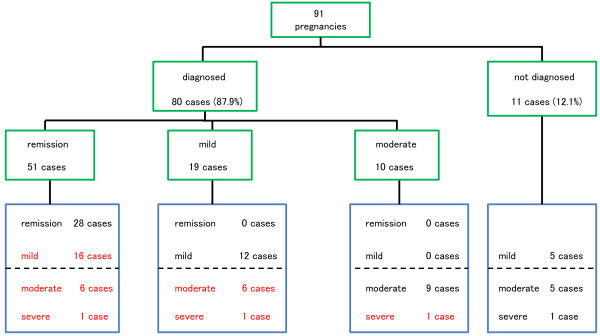
**Schema of the severity at the onset of pregnancy and change in severity during pregnancy.** Green squares indicate UC diagnosis and status at the onset of pregnancy, and blue squares indicate UC status during pregnancy. Cases experiencing exacerbation are shown in red.

With approval by the ethics committee of each institution (Nagoya University Graduate School of Medicine, Meitetsu Hospital, and Nagoya First Red Cross Hospital), we retrospectively examined 91 pregnancies in 64 UC patients (Table 1, Figure 1) experienced at our hospital and related institutions from 1991 to 2011, focusing on the relation between the progression of UC during pregnancy, the course of the pregnancy itself, and the treatment of UC. At each institution, when a UC patient became pregnant, her doctor reported her name for addition to our list. We investigated the effects of pregnancy on the course of UC by reviewing the medical records of the patients entered into the list.

**Table 1:** Characteristics of patients

	**Sixty-four patients**			**Ninety-one pregnancies (%)**
Mean ± SD		Outcome of pregnancy	normal birth	73 (80.2)
Age at diagnosis (years)	23.95±5.56		premature birth	2 (2.2)
Disease duration (years)	7.22±6.21		spontaneous abortion	8 (8.8)
Age at pregnancy (years)	32.25±4.36		therapeutic termination	8 (8.8)
Extent of UC n (%)		Receiving treatment for UC		
Total colitis	30 (46.9)	at the onset of pregnancy	total (n=80)	50 (62.5)
Left-side colitis	22 (34.4)		salazosulfapyridine (SASP)	20 (40)
Proctitis	12 (18.7)		5-aminosalicylic acid	
Clinical course n (%)			compound (5-ASA)	19 (38)
Relapsing-remitting type	53 (82.8)		SASP+prednisolone (PSL)	5 (10)
Cronic continuous type	6 (9.4)		5-ASA+PSL	3 (6)
Acute fulminating type	1 (1.6)		5-ASA+azathioprine	1 (2)
First attack type	4 (6.2)		PSL	2 (4)

### Study protocol

The medical records of the patients were reviewed by a primary investigator (M.U.), who graded the severity of UC according to the criteria of the Truelove-Witts index [19], namely as remission, mild, moderately severe (moderate), and severe. Assessment was done at the onset of pregnancy and at the worst point during the pregnancy. Exacerbation of UC was categorized as a further worsening of severity from that at the onset of pregnancy. The first trimester was considered the first 14 weeks of pregnancy, the second trimester from week 15 to 27, the third trimester from week 28 until birth, and the postnatal period was the first 8 weeks following birth.

### Statistical analysis

Statistical analysis was performed using the chi-square test or Fisher’s exact test using SPSS Statistics Version 19 (Chicago, IL). Odds ratio (OR) and 95% confidence interval (CI) were estimated when appropriate. All P values were two-tailed, and significance level was set at P<0.05.

## Results

### Patients

Patient characteristics are shown in Table 1. Among the 91 pregnancies, UC had already been diagnosed at onset in 80 cases, of which 51 were in remission, 19 were mild, 10 were moderately severe, and no cases were severe (Figure 1).

### Course of pregnancy

**Figure 2:**
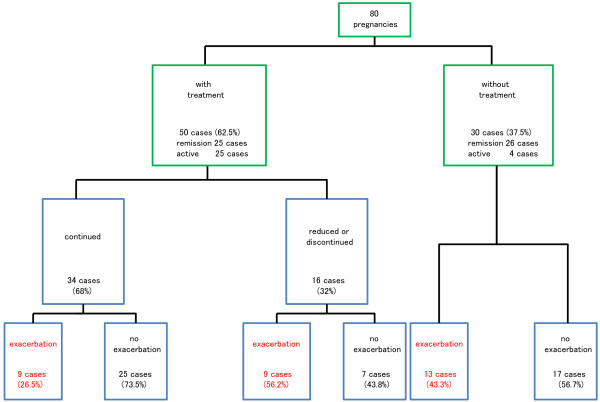
**Schema of the frequency of exacerbation of UC during pregnancy by approach to treatment.** Green squares indicate UC treatment at the onset of pregnancy, and blue squares indicate UC treatment during pregnancy. Cases experiencing exacerbation are shown in red.

Among the 80 pregnancies in which UC had been already diagnosed at the onset of pregnancy, exacerbation of UC was identified in 31 (23 of 51 in remission at onset of pregnancy (45.1%), and 8 of 29 with active disease at onset (27.6%)) (Figures 1 and 2, Table 2). When we examined the ratio of cases in the moderate or severe active stage at the worst point during pregnancy, 7 of 51 cases (13.7%) were in pregnancies in patients in remission at the onset of pregnancy, whereas 17 of 29 (58.6%) were in pregnancies in patients with active disease at the onset of pregnancy; hence, moderate or severe active stage disease at the worst point was significantly more frequent in pregnancies in the active stage at the onset of pregnancy (OR 8.9: 95%CI 3.0~26.4; P<0.01) (Table 2). With regard to the period of exacerbation of UC, 24 cases experienced exacerbation during the first trimester, 8 during the second, 3 during the third, and 7 during the puerperal period (not including second cases of exacerbation in the same patient), with the first trimester and puerperal period together accounting for 73.8%. Outcome of the 91 pregnancies was as follows: 73 normal births (80.2%), 2 premature births (2.2%), 8 miscarriages (8.8%), and 8 abortions (8.8%). Miscarriages and abortions occurred in 5 of the 51 pregnancies (9.8%) in patients in remission at the onset of pregnancy, and in 9 of the 29 pregnancies (31.0%) with active stage disease at the onset of pregnancy (OR 4.1: 95%CI 1.2~13.9; P=0.02) (Table 2).

**Table 2:** Incidence of exacerbation and miscarriages or abortions during pregnancy

**At the onset of pregnancy**	**Remission**	**Active**	**OR**	**95%CI**	**P value**
	**n=51 (%)**	**n=29 (%)**			
During pregnancy					
Exacerbation	23 (45.1)	8 (27.6)	0.46	0.2~1.2	N. S
Exacerbation to severe or moderately severe	7 (13.7)	17 (58.6)	8.9	3.0~26.4	<0.01
Miscarriage or abortion	5 (9.8)	9 (31)	4.1	1.2~13.9	0.02

### UC treatment during pregnancy

**Figure 3:**
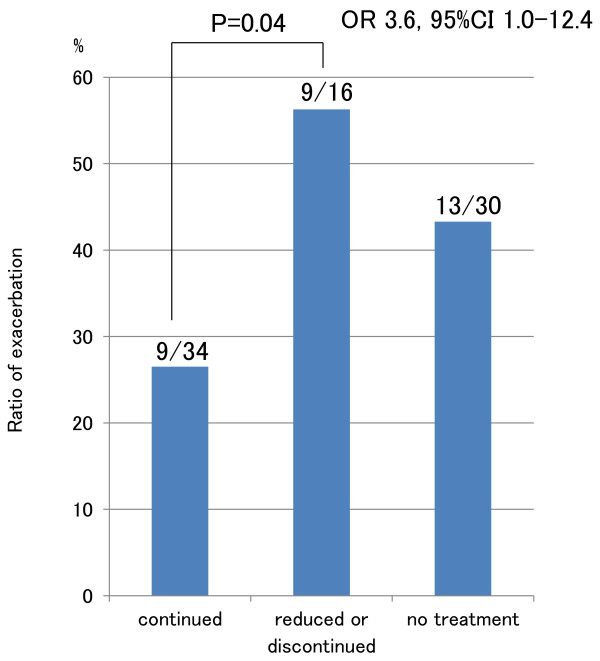
**Exacerbation rate of UC by approach to treatment.** Exacerbation rate was significantly higher in patients whose doses were reduced or whose treatment was discontinued than in those who continued pharmaceutical treatment.

**Figure 4:**
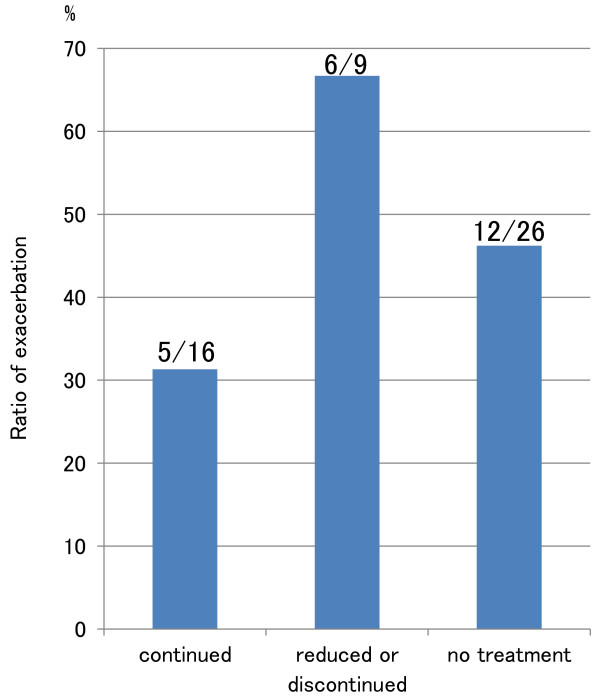
Exacerbation rate in patients in remission at the onset of pregnancy by approach to treatment.

Among the 80 cases who had already been diagnosed with UC at the onset of pregnancy, 50 (62.5%) were receiving treatment for UC at the onset of pregnancy (Table 1, Figure 2), consisting of salazosulfapyridine (SASP) in 20, 5-aminosalicylic acid compound (5-ASA) in 19, SASP and corticosteroids in 5, 5-ASA and corticosteroids in 3, corticosteroids only in 2, and 5-ASA and azathioprine in 1. Sixteen of the 50 cases (32.0%) receiving treatment for UC at the onset of pregnancy were treated with a decrease in dosage or cessation of treatment during pregnancy. Regarding outcome, exacerbation of UC was found in 9 of 34 pregnancies (26.5%) in which the same treatment was maintained during pregnancy, and in 9 of 16 pregnancies (56.3%) in which doses were reduced or treatment was discontinued (OR 3.6: 95%CI 1.0~12.4; P=0.04), while 13 of 30 cases (43.3%) that had not been treated at the onset of pregnancy experienced exacerbation (Figures 2, 3). Next, we compared UC course among patients whose UC had been in remission at the onset of pregnancy. For the 25 pregnancies in patients who had been receiving treatment and were in remission at the onset of pregnancy, exacerbation of UC occurred in 5 of 16 pregnancies (31.3%) in which the same treatment was continued, and in 6 of 9 pregnancies (66.7%) in which doses were reduced or treatment was discontinued during pregnancy; while for the 26 pregnancies in patients who had not been receiving treatment and were in remission at the onset of pregnancy, exacerbation of UC occurred 12 pregnancies (46.2%) (Figure 4). Patients in whom doses had been reduced or treatment had been discontinued were thus most likely to experience exacerbation, albeit that the differences were not significantly different.

### Patients with newly developed UC

Regarding the 11 patients who newly developed UC during pregnancy, severity was mild in 5 (45.5%), moderate in 5 (45.5%) and severe in 1 (9%) (Figure 1). Five cases were proctitis (45.5%) and six were pancolitis (54.5%). Most occurrences were identified during the early period of pregnancy, with six occurring during the first trimester (54.5%).

### Newborns status

Deformities and complications in newborns were identified in 2 of 75 births (2.7%), including 1 case of cleft palate. This patient was in remission at the onset of pregnancy, experienced exacerbation to moderate active stage disease during the first trimester, but had a normal delivery. The baby of the second patient, who was also in remission at the onset of pregnancy but did not experience exacerbation, showed prolonged jaundice but also had a normal delivery. Both patients were treated with 5-ASA during pregnancy.

## Discussion

Here, we found that the frequency of exacerbation to the moderate or severe active stage during pregnancy in patients who were in the active stage at the onset of pregnancy was significantly higher than in those who had been in remission at the onset of pregnancy. Further, patients who were treated by a decrease in dosage or cessation of treatment during pregnancy showed a significantly higher exacerbation ratio than those in whom treatment at the onset of pregnancy was maintained during pregnancy. These findings suggest that women with UC who wish to conceive should be advised to do so while in remission rather than in the active stage, and continue appropriate treatment during pregnancy.

Exacerbation rates of UC were 45.1% for patients in remission at the onset of pregnancy. Alstead [20] has shown that if conception occurs during a period of remission, about one-third of patients with IBD will relapse during pregnancy. In our study, the exacerbation ratio of patients in remission at onset tended to be higher than in their report, however, the exacerbation of UC in remission was mostly to the mild active stage. The frequency of exacerbation to the moderate or severe active stage during pregnancy in patients who were in the active stage at the onset of pregnancy was significantly higher than in those who had been in remission at the onset of pregnancy (58.6% vs 13.7%). We therefore propose that patients with UC who wish to become pregnant should do so when they are in remission.

With regard to the timing of exacerbation, 57.1% of our cases with exacerbation occurred during the first trimester. Miller [21] also reported that exacerbation was most likely to occur in the first trimester. We assume that exacerbation during this period is caused by stress, such as morning sickness. Moreover, many of our patients had a decrease in dosage or cessation of treatment during the first trimester, and we consider that some women are reluctant to receive medication during pregnancy. Ferrero et al. [22] emphasized that the exacerbation of IBD during pregnancy, particularly during the first trimester, is often due to the discontinuation of maintenance medication. These findings highlight the need for careful observation of UC patients during the first trimester.

The outcome of pregnancy in this series was normal birth in 80.2%, miscarriage or abortion in 17.6% and congenital malformation in 1.1%. Naganuma et al. [15] reported rates of 82.9% for live birth, 16.2% for spontaneous and therapeutic abortion, and 1.3% for congenital malformation in Japanese patients with UC. These results are similar to those of our study. In contrast, Hanan et al. [23] reported rates of 76%-97% for normal birth, 1%-13% for miscarriage, and 0%-3% for stillbirth. They also observed congenital abnormalities in 0%-3%, which was not significantly different from that of the non-UC controls. Recently Bortoli et al. [24] reported rates of 94.7% for normal birth and 5.3% for miscarriage or abortion, which were also not significantly different from that of the non-UC controls. In our study, miscarriage or abortion occurred in 9.8% of pregnant UC patients who had been in remission at the onset of pregnancy versus 31% of those who had been in the active stage at onset, and was therefore significantly higher among patients in the active stage at the onset of pregnancy. The tendency of miscarriage or abortion appeared to decrease with time, indicating the need for a prospective study of pregnant women with UC in Japan.

Our study identified 11 occurrences of newly arising UC during pregnancy. Onset was most frequent during the first trimester, and severity was mostly moderate or severe. Willoughby et al. [25] reported that 8 of 16 patients had their first attack of UC in the first trimester, and that the first attack in the 3 of 4 patients with severe disease occurred in the first trimester. These findings suggest that patients with the new onset of UC during pregnancy tend to then experience exacerbation to a more severe degree, and accordingly require more stringent therapy.

In this study, 62.5% of patients were under pharmaceutical treatment at the onset of pregnancy. Drugs such as SASP, 5-ASA and corticosteroids are categorized as Food and Drug Administration (FDA) pregnancy-category-B drugs, and as safe drugs by the European Panel on the Appropriateness of Crohn's Disease Therapy (EPACT) and the ECCO [8,10]. In Japan, the Ministry of Health, Labour and Welfare has declared immunoregulators to be contraindicated for use in pregnant women, and we found only one patient who used an immunoregulator for UC during pregnancy in our study. This patient became pregnant in remission, did not experience exacerbation during pregnancy, and gave birth without complications. The ECCO classifies azathioprine and 6-mercaptopurine as safe [10]. The EPACT classifies these drugs as having equivocal safety during the first trimester and nursing, and as safe drugs during the second and third trimesters [8], while the FDA classifies them as category-D. These reports suggest that immunoregulators might be safe, but should be used cautiously in patients with UC who become pregnant with full discussion of the advantages and risks of their use.

The exacerbation rate in UC patients for whom doses were reduced or treatment was discontinued during pregnancy was significantly higher than in those who maintained the same pharmaceutical treatment as before pregnancy. Among patients in the three groups who were in remission at the onset of pregnancy, the lowest exacerbation rate was seen in patients who maintained the same pharmaceutical treatment while the highest rate was seen in patients for whom doses were reduced or treatment was discontinued during pregnancy, albeit that the differences were not statistically significant owing to the small sample size and limited statistical power of this study (Figure 4). In their meta-analysis of pregnancy outcomes in women with IBD following exposure to 5-ASA (including SASP) drugs, Rahimi R et al. [26] reported a no greater than 1.14-fold increase in spontaneous abortion, and a 1.16-fold increase in congenital abnormalities. It is safe and important to continue proper maintenance therapy during pregnancy.

Several limitations of our study warrant mention. The retrospective design of the study may have introduced a degree of selection bias. Because we collected our data from medical records, we were unable to verify all information about the mode of delivery and exact weight of babies. Comparisons were also hampered by the range of patient treatments and instructions among institutions and doctors. Further prospective studies with an increased number of pregnant UC patients are therefore required.

## Conclusions

This study shows that the impact of UC on the progress of pregnancy does not widely differ between Japanese and Western patients. UC patients wishing to become pregnant should be advised to do so while they are in the remission stage, and to continue appropriate pharmaceutical treatment during pregnancy.

## Abbreviations

UC: Ulcerative colitis; OR: Odds ratio; CI: Confidence interval; ECCO: European Crohn's Colitis Organization; FDA: Food and Drug Administration; EPACT: European Panel on the Appropriateness of Crohn's Disease Treatment.

## Competing interest

The authors declare that they have no conflict of interest.

## Authors’ contributions

MU, KM, MM, YH, KM, RM, NO, TY, and JH participated in the acquisition of patients’ data. TA, OW, and YN participated in the design of the study. MU, TA, KI, OM, and OW performed the statistical analysis and interpreted the data. TA and MU drafted the manuscript. KI made critical revision of the manuscript for important intellectual content. HG and TA supervised the study. All authors read and approved the final manuscript.
